# A Digital Health Approach to Improve Compliance With Surveillance Colonoscopy Guidelines: The SCOPES Program: Study Protocol for a Stepped‐Wedge Cluster Trial

**DOI:** 10.1002/cam4.71456

**Published:** 2026-02-03

**Authors:** Erin L. Symonds, Geraldine Laven‐Law, Isabelle Keel, William Wilson, Lyle J. Palmer, Muktar Ahmed, Kalindra Simpson, Chetan Pradhan, Rajvinder Singh, Quentin Ralph, Ilmars Lidums, William Tam, Paul Hollington, Charles Cock, Phil Worley, Jean M. Winter, Graeme P. Young, SCOPES Advisory Committee, Billie Bonevski, Billingsley Kaambwa, Devinder Raju, Eleonora Feletto, Finlay Macrae, Ganessan Kichenadasse, Grace O’Donohue, Heath Milne, Hooi Ee, Jayne Sandford, Kathryn Cornthwaite, Luke Betts, Mark Schoeman, Michelle Coats, Molla M. Wassie, Norma Bulamu, Oliver Frank, Peter Pascoe, Richard Reed, Richard Woodman, Robert Fraser, Sally Knuckey, Sharon Gillespie, Sharyn Coles

**Affiliations:** ^1^ Department of Gastroenterology, Surgical and Perioperative Medicine, Southern Adelaide Local Health Network Flinders Medical Centre Bedford Park South Australia Australia; ^2^ Flinders Health and Medical Research Institute, College of Medicine and Public Health Flinders University Bedford Park South Australia Australia; ^3^ Department of Anaesthesia, Northern Adelaide Local Health Network Lyell McEwin Hospital Elizabeth Vale South Australia Australia; ^4^ School of Public Health, Faculty of Health and Medical Sciences University of Adelaide Adelaide South Australia Australia; ^5^ Australian Institute for Machine Learning University of Adelaide Adelaide South Australia Australia; ^6^ Department of Surgery, Riverland Mallee Coorong Local Health Network Riverland General Hospital Berri South Australia Australia; ^7^ Department of Gastroenterology, Northern Adelaide Local Health Network Lyell McEwin Hospital Elizabeth Vale South Australia Australia; ^8^ School of Medicine University of Adelaide Adelaide South Australia Australia; ^9^ Department of Surgery, Eyre and Far North Local Health Network Port Lincoln Hospital Port Lincoln South Australia Australia; ^10^ Department of Gastroenterology, Central Adelaide Local Health Network The Queen Elizabeth Hospital Woodville South Australia Australia; ^11^ Department of Gastroenterology, Central Adelaide Local Health Network The Royal Adelaide Hospital Adelaide South Australia Australia; ^12^ Department of Surgery, Surgical and Perioperative Medicine, Southern Adelaide Local Health Network Flinders Medical Centre Bedford Park South Australia Australia

**Keywords:** algorithm, colonoscopy recall guidelines, colorectal cancer, natural language processing, neoplasia, patient‐reported measures, post‐polypectomy, registry, surveillance

## Abstract

**Introduction:**

Individuals at elevated risk of developing colorectal cancer (CRC) benefit from regular surveillance colonoscopies. However, many countries lack well‐managed recall processes, leading to either excessive or insufficient colonoscopy use, both of which have significant consequences. A nurse‐coordinated surveillance program has been shown to improve compliance with surveillance guidelines but is associated with a costly administration burden. This study aims to create a multicenter, stepped‐wedge cluster trial that will integrate digital processes into this model to optimise colonoscopy management, reduce resource burden and ensure equitable service delivery across multiple healthcare sites.

**Methods:**

Data from colonoscopy and pathology reports will be extracted into a clinical registry and natural language processing will be used to structure the data. Rule‐based algorithms (based on the Australian colonoscopy surveillance guidelines (but adaptable to other international standards), and with version control) will assess the need for future surveillance colonoscopies and recommend appropriate follow‐up intervals. The accuracy of the recommendations will be evaluated by nurse coordinators, with adherence to the guidelines assessed both at baseline and 6 months post‐implementation. Patient‐reported measures will be collected before and during trial implementation to assess satisfaction with the surveillance processes. Outcome measures will include evaluation of guideline compliance, key performance indicators for the quality of endoscopic services and cost‐effectiveness.

**Discussion:**

This trial will establish the performance, acceptability and cost‐effectiveness of a digital health approach to managing surveillance colonoscopy. This will improve healthcare delivery by providing a cost‐effective way to manage colonoscopy demand and to mitigate risk for CRC.

## 1 Introduction

Colorectal cancer (CRC) is the second leading cause of cancer‐related death worldwide, with almost 1 million deaths reported in 2020 [1]. The incidence is expected to rise to 3.2 million cases per year by 2040 [2], highlighting the need for effective screening and surveillance strategies. Colonoscopy is essential to CRC prevention, enabling early detection and removal of precancerous lesions [3, 4, 5]. Regular surveillance colonoscopy is recommended for individuals at an elevated risk for CRC, such as those with prior adenomas, CRC, or a significant family history [6, 7, 8]. However, the increasing demand for colonoscopies [9, 10] places a significant burden on healthcare resources, especially when surveillance is performed too frequently [11, 12].

Surveillance colonoscopies can account for over one‐third of all procedures [13], with guidelines recommending intervals, based on previous findings, patient age, health status and examination [6, 14]. Guidelines for surveillance are complex and differ across the world, making adherence challenging [15, 16, 17]. Studies show that up to two‐thirds of surveillance colonoscopies occur at inappropriate intervals [15, 16, 18, 19, 20, 21] either too early, increasing risks and costs [22, 23], or too late, risking cancer progression [24].

Many systems rely on a multi‐step referral process involving specialists and family physicians, which risks patient loss to follow‐up. Furthermore, few clinical practices record patient‐reported measures to determine acceptability with the surveillance process. The Southern Co‐operative Program for the Prevention of Colorectal Cancer (SCOOP), a nurse‐coordinated registry in Australia (commenced in 1999), has demonstrated improved adherence to national guidelines (from 37% to 96% [25, 26]), reduced unnecessary procedures [27] and long‐term compliance [28, 29]. However, manual data review and collection for surveillance recommendations are resource‐intensive.

Digital automation has shown promise in reducing administrative burden and improving accuracy, with a recent study demonstrating 98.7% accuracy in automated surveillance recommendations for post‐polypectomy cases [30]. Automation and natural language processing (NLP) are increasingly used in endoscopy to extract quality indicators such as adenoma and serrated polyp detection rates from procedure reports [31, 32]. While these developments highlight the potential for digital health to support endoscopy, the SCOPES program specifically applies rule‐based algorithms to optimise surveillance interval recommendations rather than real‐time quality monitoring. Expanding this approach to broader risk groups within a nurse‐coordinated model may improve efficiency, reduce costs and enhance compliance with surveillance guidelines.

## 2 Objective

The current limitations in the provision of surveillance colonoscopies include: (1) limited capacity for increased colonoscopy demand, (2) poor compliance with surveillance guidelines, (3) disparities in quality of care between different providers and regions and (4) lack of large volume data registries to allow for research into optimising colonoscopy processes and preventing CRC. This study will apply the proven framework of a nurse‐coordinated CRC surveillance model and integrate this with digital processes to create a new coordinated care program to manage surveillance colonoscopies and provide cost‐effective service equity across multiple healthcare sites.

## 3 Methods

SCOPES (Surveillance for Colorectal Cancer Prevention) is registered with the Australian New Zealand Clinical Trials Registry (number: ACTRN12624001429549, registered 6 December 2024). This study was approved by the Southern Adelaide Clinical Human Research Ethics Committee (SAC HREC), Adelaide, South Australia (HREC approval number 2024/HRE00165).

Patients and members of the public contributed to the co‐design of participant communications and survey materials to ensure clarity and accessibility.

### 3.1 Study Setting and Design

The SCOPES program will provide automated recommendations for colonoscopy surveillance intervals, which align with the timings recommended in the Australian guidelines [6], for people at elevated risk for developing CRC across five South Australian local hospital networks (inclusive of 8 hospital sites). The process will be overseen by a nurse‐coordinated team, with the digital processes streamlining existing clinical resources, using natural language processing to convert reports to structured data and provide appropriate algorithm‐based surveillance recommendations. These recommendations will be validated against existing surveillance colonoscopy data from the nurse‐led SCOOP program which has been well annotated, audited and cleaned over the years, with reviews conducted by multiple experts. These data were collected manually for a range of elevated risk indications related to personal and familial factors.

Hospital audits and patient‐reported measures will determine compliance with national guidelines for surveillance colonoscopy and consumer satisfaction before program implementation. The program will be implemented in one hospital network at a time, across five different hospital networks, and evaluated using a stepped‐wedge cluster trial design. Audits will be repeated after at least 6 months of implementation. Feasibility assessments will determine the accuracy, acceptability and cost‐effectiveness of the surveillance program compared to current practices.

### 3.2 Inclusion and Exclusion Criteria

All colonoscopies conducted at participating hospitals will be included (Table 1), regardless of indications and patient risk factors. Individuals who opt out of having their health data audited will be excluded from the study. The hospital coverage captures approximately 50% of the colonoscopies completed in South Australia, and > 80% of procedures completed in government‐run public hospitals [33].

**TABLE 1:** South Australian public hospitals implementing the integrated nurse and digital surveillance program.

Locality	Hospital network	Included hospitals
Adelaide metropolitan	Southern Adelaide Local Health Network	Flinders Medical Centre Noarlunga Health Services
	Northern Adelaide Local Health Network	Lyell McEwin Hospital Modbury Hospital
	Central Adelaide Local Health Network	The Queen Elizabeth Hospital The Royal Adelaide Hospital
Regional	Riverland Mallee Coorong Local Health Network	Riverland General Hospital
	Eyre and Far North Local Health Network	Port Lincoln Hospital

### 3.3 Colonoscopy Standards

Colonoscopies scheduled within participating hospital networks across South Australia will be conducted according to best practice and accreditation requirement by trained endoscopists. The quality of the bowel preparation will be evaluated using the Boston Bowel Preparation Scale [34]. Intubation will be considered complete if the caecum, terminal ileum, or anastomosis (for patients with a prior colonic resection) is reached. Colonoscope withdrawal time from caecum to rectum will be recorded. Histopathology‐confirmed adenoma and sessile serrated lesion detection rates will be recorded for each proceduralist, following guidelines [6, 35].

### 3.4 Baseline Site Assessments

The baseline efficiency of each hospital's colonoscopy surveillance process will be determined by calculating the staffing time burdens and costs for surveillance colonoscopy, as well as the appropriateness of each given surveillance interval, and time taken from colonoscopy until the patient receives communication regarding their next interval recommendation.

As per usual hospital practice, the next recommended colonoscopy surveillance interval will be provided to the patient and their family physician by the clinical team after colonoscopy. Compliance rates will be compared to the Australian guidelines for surveillance colonoscopy [6], and the time for each patient and their family physician to receive communication regarding the surveillance interval will be determined from medical records in a random selection of 10% of the total colonoscopies completed in 1 year (5% performed for surveillance and 5% for non‐surveillance indications) per hospital. Staffing time to provide the recommendations and communications will be prospectively determined for a minimum of 50 individuals undergoing surveillance colonoscopies at each hospital, with the costs calculated through consideration of time and salary rates. Clinical teams that are involved with the surveillance processes (proceduralists, nurses, administration and family physicians) will be interviewed to collect qualitative data on acceptability and limitations of current practices.

Surveys will be collected from a random selection of 100 patients per hospital before and after their surveillance colonoscopy, capturing patient demographics, assessing colonoscopy experience, quality of life and the quality of endoscopic services provided. Quality of life will be determined using the EQ‐5D‐5L validated measure [36].

Key performance indicators for the quality of endoscopic services (using recommendations provided in several consensus reports [6, 37, 38]; Table S1) will be calculated for a period of at least 6 months.

### 3.5 Automated Clinical Data Collection

Relevant data (Table 2) will be sourced from each hospital's existing clinical databases. Clinical data provided in structured fields, such as in colonoscopy reports and medical records, will be cleaned and collated into relevant fields within a clinical registry in the digital platform (computer software). Relevant elements from synoptic reports and unstructured or free‐text clinical data, such as those used in histopathology and surgical reports, will be extracted and cleaned through the spaCy library in Python for natural language processing (NLP) and rule‐based regular expressions. The NLP system will account for synonyms and variations in terminology, allowing differentiation between key terms such as ‘no high‐grade dysplasia’ versus ‘high‐grade dysplasia’. The cleaned data (Table 2) will then be collated within the digital platform.

**TABLE 2:** Demographic and clinical data variables required for generating automated colonoscopy surveillance intervals.

Category	Data source	Variables collected
Demographics	Electronic medical record	Patient identifiers Age Sex Body measurements Postal address Family physician contact details Family history of colorectal cancer or genetic syndrome Personal risk factors (e.g., genetic syndromes or past colorectal cancer or neoplasia)
Colonoscopy provision	Colonoscopy reporting system	Date of procedure Proceduralist Indication for colonoscopy
Colonoscopy quality	Colonoscopy reporting system	Intubation distance Withdrawal time Bowel preparation score
Lesion detection	Colonoscopy reporting system Histopathology reports Surgery reports	Number of lesions Lesion morphology Lesion size Anatomical location Removal technique and completeness Lesion histology Presence and extent of dysplasia or invasive cancer
Other clinical factors	Electronic medical record, Colonoscopy reporting system	Comorbidities or other relevant colonoscopic observations Medications
Adverse events	Electronic medical record, Colonoscopy reporting system	Post‐polypectomy bleeding Bowel perforation Hospitalisation Death

### 3.6 Automation of Surveillance Recommendations Using Computer Algorithms

Following data collation into the clinical registry, rule‐based algorithms will provide automated recommendations for colonoscopy surveillance intervals using the patient, colonoscopy and histopathology data. Factors considered within the algorithm will include the date and quality of the colonoscopy procedure, findings from the two previous colonoscopies, age, body mass index, comorbidities that are relevant to calculating the Charlson Comorbidity Index, personal and family history of a higher risk factor including CRC, past significant neoplasia, serrated polyposis or a hereditary CRC syndrome (e.g., Lynch syndrome).

Algorithms will be primarily rule‐based, using hierarchical prioritisation. The hierarchy of criteria will be classified based on Australian clinical guidelines [6], rather than unsupervised clustering, and then integrated into recommender systems. The ruleset will be frozen, version‐controlled and include explicit conflict resolution and edge‐case handling. Risk factors considered for surveillance recommendations that align with the current Australian clinical practice guidelines are provided in Table S2.

### 3.7 Validation of Digital Processes

The efficacy of data extraction, cleaning and collation, as well as algorithm accuracy will be validated in a random selection of at least 5000 archival colonoscopy reports and corresponding histopathology outcomes, which have been manually recorded as part of the long‐running SCOOP program [29], allowing testing of at least 20% of all archival data. Each colonoscopy captured as part of the SCOOP program has been manually entered into an electronic database. This manually curated SCOOP dataset will serve as the gold standard for NLP performance benchmarking. Independent assessors performing manual review will be blinded to the algorithm's output to minimise bias. All surveillance recommendations within the program have been provided by trained nurses, certified by the treating clinicians, with their accuracy audited by data scientists.

Eighty percent of the SCOOP dataset will be used for the SCOPE's digital process algorithm development and parameter tuning, and the remaining 20% will be used as a hold‐out validation dataset. Comparisons between manual and automated reporting will be performed using the SCOOP dataset as the reference standard, with adjustments made to the algorithm's parameters to ensure maximal concordance. A prospective external validation will be performed at the first implementation site prior to feedback to end users, to evaluate real‐world performance and minimise overfitting risk. Prespecified criteria for algorithm update vs. lock, including intercept/slope recalibration, will be applied while maintaining the original ruleset unchanged. All discrepancies will be manually reviewed independently by at least two team members, with the most probable reason for discrepancy identified and adjusted for algorithm improvement. Performance will be evaluated using standard NLP metrics. Precision of the digital process to measure the overall proportion of correct interval predictions will be defined as the proportion of true positive predictions; recall will gauge the model's ability to identify true positives, with the F1 score calculated to determine predictive performance. Field‐by‐field performance will be reported separately for all key variables (bowel prep quality, histology, dysplasia status and surveillance interval), distinct from overall surveillance‐interval concordance, in line with recent gastrointestinal‐specific NLP reporting standards [39].

### 3.8 Implementation of the Digital Process

This trial will compare a control phase (usual practice) with an intervention phase (the nurse‐coordinated program integrated with digital processes [i.e., SCOPES]) using a stepped‐wedge cluster trial design [40]. Implementation will commence in the second year of the study, where one new hospital network will have the intervention implemented every 6 months, until all five included South Australian hospital networks receive the intervention. The stepped‐wedge design can provide greater statistical power than a randomised parallel group cluster trial design under certain conditions, such as moderate intra‐cluster correlation (ICC), adequate cluster size and multiple steps, with fewer time constraints in the introduction of the digital processes [41]. Each hospital network is expected to perform up to 10,000 colonoscopies per year and will receive the intervention phase for at least 12 months.

**FIGURE 1:**
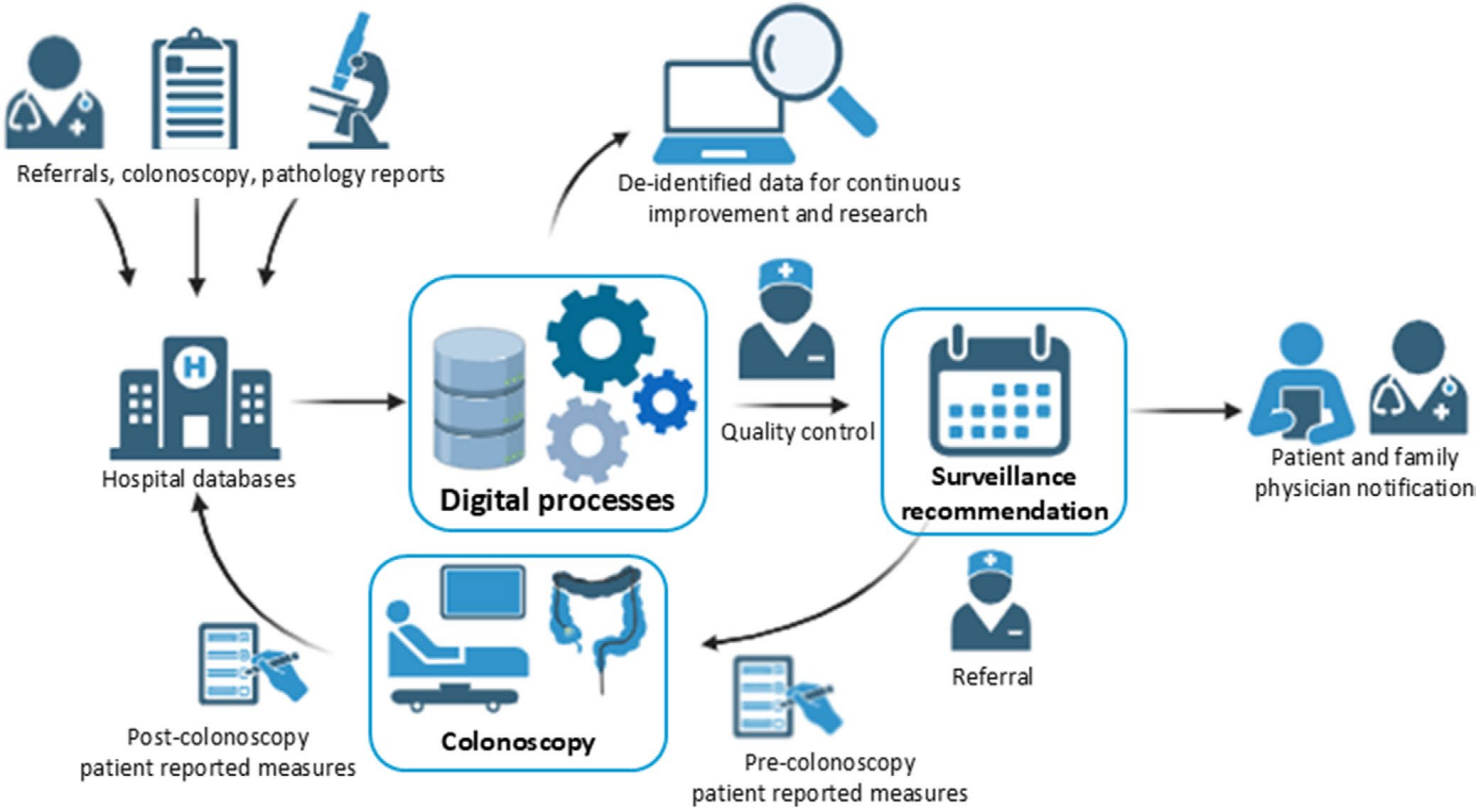
A digital healthcare approach for automated colonoscopy surveillance recommendations, integrated with a nurse‐coordinated program.

Figure 1 provides an overview of the digital processes. Following the automatic upload of colonoscopy and associated pathology data into the clinical registry, the SCOPES algorithm will provide a surveillance recommendation. Each recommendation will be reviewed and approved by a trained nurse. In cases where the nurse and algorithm identify discrepant surveillance interval recommendations, conflicts will be resolved by a third‐party nurse or proceduralist as appropriate. All reasons for discrepant surveillance intervals will be recorded for quality improvement purposes.

Patients and family physicians will be informed of the prescribed surveillance interval and when the next surveillance colonoscopy is due, with the timing from colonoscopy until this communication recorded. Online surveys capturing the same patient‐reported measures collected at baseline will also be offered before and after surveillance colonoscopy, commencing at each hospital, 6 months after implementation of the intervention. In addition, interviews will be conducted with the clinical teams involved in the surveillance processes (proceduralists, nurses, administration and family physicians), to gauge end‐user acceptability. Measures of the key performance indicators for the quality of endoscopic services (Table S1) will be repeated at least 6 months after implementation of the intervention at each site.

### 3.9 Data Governance and Safety Monitoring

All study data will be stored on secure institutional servers compliant with role‐based access control, multifactor authentication and encrypted data transmission between participating sites. Automated audit logs will record all data modifications and algorithm interactions. To mitigate automation bias, nurse coordinators will review and confirm all algorithm‐generated recommendations using a structured checklist before acceptance into the patient record. A data and safety monitoring plan will oversee potential harms such as delayed colonoscopies or missed advanced neoplasia.

### 3.10 Outcome Parameters

All study outcomes will be compared between baseline and post‐intervention measures. The primary outcome is the proportion of automated interval recommendations for surveillance colonoscopy that match those derived from a prespecified ruleset directly based on the Australian guidelines. This ruleset serves as the gold standard for interval assignment and is explicitly programmed from the most recent national surveillance criteria. Algorithm outputs will be independently compared against this reference ruleset, with nurse recommendations serving as a secondary comparator. Validation will be conducted blinded to algorithm outputs to avoid bias, and any discrepancies between algorithm, ruleset and clinical recommendations will be adjudicated by an independent gastroenterologist panel.

Secondary outcomes include:

- The timing of the scheduled colonoscopy in relation to the recommended timeframe, with ‘on‐time’ colonoscopy defined as occurring within ±6 months of the due date specified by guideline recommendations.
- The time taken to review the colonoscopy outcomes and communicate the prescribed surveillance interval with the patient and family physician.
- The prevalence of individuals lost to follow‐up.
- The prevalence of patients with acceptable bowel preparation quality, complete intubation and adequate withdrawal time.
- The incidence rate for advanced neoplasia (with hierarchical classification prioritising invasive CRC > stage 0 CRC > advanced adenoma), including CRC, adenomatous polyps or sessile serrated lesions ≥10 mm in size, with villous change, or with high‐grade dysplasia, sessile serrated lesions with any dysplasia and any traditional serrated adenoma.
- The incidence rate for adverse events (e.g., bleeding, perforation, hospitalisation and death).
- Acceptability of the intervention to patients and clinical teams.
- Cost‐effectiveness of the intervention.

Among the secondary outcomes, those relating to colonoscopy quality metrics and timeliness are confirmatory and prespecified, whereas patient‐reported measures and thematic feedback analyses are exploratory.

Trial findings will be reported according to the CONSORT‐AI extension guidelines [42].

### 3.11 Sample Size and Power

The stepped‐wedge cluster randomised trial includes five local health networks (cluster) with eight participating hospitals, contributing an average of 1417 colonoscopy episodes per 3‐month period (adjusted for 10% attrition) across seven sequential periods (one baseline and six rollout steps). Assuming an ICC of 0.01, a baseline guideline concordance of 60%, and an expected post‐implementation improvement to 75% (15% absolute increase), the design archives approximately 90% power at a two‐sided *α* = 0.05 under a binomial mixed‐effects model. Power was estimated via simulation (*n* = 1000 iterations per effect size), incorporating cluster and period fixed effects. A power curve (Figure S1) illustrates sensitivity to effect size assumptions, a stepped‐wedge design diagram (Figure S2) depicts the rollout sequence, and a summary of power estimates across effect sizes is presented in Table S3.

For the patient‐reported outcomes, assuming eight hospitals with paired before and after implementation (100 participants per hospital per period, total *N* = 1600), we estimate > 80% power (*α* = 0.05, two‐sided) to detect a small‐to‐moderate standardised mean difference (Cohen's *d* ≈ 0.25–0.30) in EQ‐5D‐5L scores, accounting for within‐cluster correlation (ICC ≤ 0.02) and within‐period correlation of repeated measures (*ρ* ≈ 0.5). This is appropriate for evaluating meaningful changes in patient‐reported measures at the hospital level.

### 3.12 Statistical Analyses

All sites will be allowed a three‐month post‐implementation period of transition before outcome measurement. Temporal confounding will be mitigated through the use of models (e.g., interrupted time series analysis) which adjust for secular trends. Given the stepped‐wedge cluster randomised design, analyses will explicitly include fixed effects for time period and intervention status, and random effects for site (cluster) to account for ICC. With only eight clusters, we will apply the Kenward–Roger correction to adjust standard errors and *p* values are not inflated due to small number of clusters. Data analysis will be conducted using Python (3.11) and R (version 4.4.1).

The percentage of surveillance recommendations that match with Australian guidelines will be calculated from audit data, and the prevalence of individuals lost to follow‐up, prevalence of those with acceptable colonoscopy quality, and incidence rates for advanced neoplasia and adverse events will be summarised using descriptive statistics. All measures will be directly compared to each site's own baseline measures in a paired manner using McNemar's tests for proportions, Wilcoxon and Kruskal–Wallis signed‐rank tests for continuous measures and mixed‐effects Poisson regression for incidence rates. Analyses will be conducted on an intention‐to‐treat basis. A prespecified generalised linear mixed model (GLMM) with a logit link will be used for the primary binary outcome (guideline concordance) including fixed effects for time period and intervention, random intercepts for clusters and adjustment for potential confounders (e.g., age, indication and baseline quality metrics). To address the small number of clusters (*K* = 5), we specify that inference will be based on cluster‐preserving parametric bootstrap for both the intervention coefficient and marginal contrasts. Multiple comparisons across secondary outcomes will be controlled using Holm–Bonferroni correction to limit type I error. Additional sensitivity analyses will include (i) cluster‐level summaries with Kenward–Roger correction, (ii) generalised estimating equations with small‐sample corrected SEs and (iii) permutation tests at the cluster level. If national guideline text changes during the trial, an indicator for the new guideline will be included, and analyses will be repeated using both old and new mapping.

Where relevant, univariate followed by multivariable modelling will be undertaken by adding variables considered clinically important or statistically significant from the univariate model to adjust for confounding effects. Hospitals will be treated as random effects, and main effects will be the intervention group; that is, before and after implementation of the intervention. Missing data will be handled using multiple imputation under the missing‐at‐random assumption. For bowel preparation quality, missing values will be imputed as ‘adequate’ in the primary analysis, reflecting routine clinical documentation where poor preparation is explicitly noted, with sensitivity analyses applying conservative rules assumption imputing missing values as ‘poor’.

Multivariable generalised linear models and pairwise comparisons will be used to assess the association between patient‐reported measures (such as satisfaction with the colonoscopy experience) and acceptance of the digital healthcare approach. Thematic analysis will be conducted to establish clinical end‐user opinions on the surveillance program. Prespecified subgroup analyses will assess intervention effects across patient age, sex, socioeconomic status (by postcode linkage) and hospital location, with multiplicity controlled via Holm–Bonferroni correction. To ensure equitable healthcare delivery, analyses will include age groups ≥ 75 years, comorbidity burden (based on the Charlson Comorbidity Index categories), socioeconomic status and regional vs. metropolitan location.

Quality of life measures will be used to calculate the incremental costs before and after implementation of the intervention per quality‐adjusted life‐year gained (QALY) in a model‐based economic evaluation. Implementation costs will be obtained from administrative data. The economic evaluation will adopt a health‐system perspective with a 12‐month time horizon, 5% annual discounting and QALY derivation from EQ‐5D‐5L value sets. Probabilistic sensitivity analyses will assess parameter uncertainty, and reporting will align with CHEERS 2022 guidelines [43]. Patients will be recruited consecutively at each site, with reminders and follow‐up strategies implemented to maximise retention. Missing responses in surveys and quality‐of‐life instruments will be addressed using multiple imputation under the missing‐at‐random assumption, consistent with the approach for clinical outcomes.

## 4 Discussion

Best practice guidelines on how frequently surveillance colonoscopy should be performed often have poor adherence [15, 16, 18, 19, 20, 21]. The increasing demand on colonoscopy services [9] lengthens waiting times, thereby increasing patient anxiety, and increasing the risk for CRC and mortality [44, 45]. There is a clear need for a reliable, efficient surveillance program to deliver timely, evidence‐based recommendations for individuals at elevated CRC risk.

This study will evaluate the integration of a digital, algorithm‐supported approach into a nurse‐led surveillance program. By reducing inappropriate procedures [15, 16, 17], this model has the potential to improve cost‐effectiveness and reduce burdens on primary and specialist care. Importantly, it also aims to address under‐servicing of high‐risk individuals.

Although the study is limited to five hospital networks in South Australia, these sites represent a mix of metropolitan and regional centers and cover over 80% of public colonoscopies in the state [33]. This focused implementation enables robust evaluation before potential national or international scale‐up. In addition, this study's strength lies in the integration within the framework of the nurse‐coordinated SCOOP surveillance program to maximise clinical efficiency. The stepped‐wedge trial design ensures practical rollout across sites. As the SCOPES program is tailored to Australian healthcare settings and guideline frameworks, further work will be needed to evaluate adaptability across international health systems and contexts before broader implementation.

## 5 Conclusion

This study will implement a digital, nurse‐coordinated model to automate data collection and streamline surveillance colonoscopy recommendations, ensuring that all patients receive evidence‐based care, and improving CRC prevention. By capturing patient‐reported outcomes and collecting high‐quality clinical data, the program also provides a platform for continuous improvement in surveillance practices.

## Author Contributions

**Erin L. Symonds:** conceptualization, methodology, investigation, resources, writing – original draft, funding acquisition, writing – review and editing, supervision. **Geraldine Laven‐Law:** writing – review and editing, methodology, visualisation, conceptualization. **Isabelle Keel:** project administration, methodology, writing – review and editing. **William Wilson:** methodology, resources, writing – review and editing, conceptualization. **Lyle J. Palmer:** methodology, writing – review and editing. **Muktar Ahmed:** methodology, writing – review and editing. **Kalindra Simpson:** conceptualization, writing – review and editing, methodology. **Chetan Pradhan:** methodology, writing – review and editing, resources. **Rajvinder Singh:** methodology, writing – review and editing, resources. **Quentin Ralph:** methodology, writing – review and editing, resources. **Ilmars Lidums:** methodology, writing – review and editing, resources. **William Tam:** methodology, writing – review and editing. **Paul Hollington:** conceptualization, methodology, resources, writing – review and editing. **Charles Cock:** writing – review and editing, methodology, conceptualization, resources. **Phil Worley:** conceptualization, methodology, writing – review and editing, resources. **Jean M. Winter:** conceptualization, methodology, writing – review and editing. **Graeme P. Young:** conceptualization, methodology, writing – review and editing, supervision.

## Funding

Medical Research Future Fund administered by the Department of Health, Disability and Ageing (Grant ID MRFCRI000173, CIA Erin Symonds). The funders have no role in the design, analysis or reporting of the trial.

## Ethics Statement

This study was approved by the Southern Adelaide Clinical Human Research Ethics Committee (SAC HREC), Adelaide, South Australia (HREC approval number 2024/HRE00165). SCOPES (Surveillance for Colorectal Cancer Prevention) is registered with the Australian New Zealand Clinical Trials Registry (number: ACTRN12624001429549, registered 6 December 2024).

## Conflicts of Interest

The authors declare no conflicts of interest.

## Supporting information


**Appendix S1:** cam471456‐sup‐0001‐AppendixS1.docx.

**File S1:** cam471456‐sup‐0002‐FileS1.docx.

## Data Availability

De‐identified registry extracts and analytic code will be made available upon reasonable request, following governance approval and institutional data‐sharing policies. Please contact the corresponding author for further details.

## Appendix

*SCOPES Advisory Committee: Billie Bonevski (Flinders University), Billingsley Kaambwa (Flinders University), Devinder Raju (Northern Adelaide Local Health Network (NALHN)), Eleonora Feletto (Daffodil Centre, the University of Sydney), Finlay Macrae (The Royal Melbourne Hospital), Ganessan Kichenadasse (Flinders University), Grace O'Donohue (Commission on Excellence and Innovation in Health (CEIH)), Heath Milne (NALHN), Hooi Ee (Sir Charles Gairdner Hospital), Jayne Sandford (Southern Adelaide Local Health Network (SALHN)), Kathryn Cornthwaite (Flinders University), Luke Betts (SALHN), Mark Schoeman (Central Adelaide Local Health Network (CALHN)), Michelle Coats (SALHN), Molla M. Wassie (Flinders University), Norma Bulamu (Flinders University), Oliver Frank (The University of Adelaide), Peter Pascoe (Consumer), Richard Reed (Flinders University), Richard Woodman (Flinders University), Robert Fraser (SALHN), Sally Knuckey (CALHN), Sharon Gillespie (CALHN) and Sharyn Coles (CEIH, Consumer).
