# Supplementary material for: A Digital Health Approach to Improve Compliance With Surveillance Colonoscopy Guidelines: The SCOPES Program: Study Protocol for a Stepped‐Wedge Cluster Trial
Source: Cancer Med. 2026 Feb 3;15(2):e71456. doi: 10.1002/cam4.71456 (PMC12868931; doi:10.1002/cam4.71456)
Supplement: Supplementary file 1 — Appendix S1: cam471456‐sup‐0001‐AppendixS1.docx. [file CAM4-15-e71456-s002.docx]

**SUPPLEMENTARY INFORMATION**

**Supplementary Table S1**. Quality elements appropriate for incorporation into a surveillance program.

| Quality element | Quality standard | Rationale | Surveillance program requirements |
| --- | --- | --- | --- |
| Bowel preparation quality^1,2^ | *“Less than 10% of patients should require a repeat colonoscopy due to poor bowel preparation”* | Poor bowel preparation is a burden on time for patients and a burden on resources due to having to repeat many of the procedures within 12 months.  A poor bowel preparation increases the risk for missing a cancer (1). This needs to be addressed through patient education and appropriately recording bowel preparation quality. | Provision of education (e.g. links to online videos) so that patients understand the importance of appropriate bowel preparation prior to colonoscopy.  All completed colonoscopies should record bowel preparation quality using an international scoring system (e.g. Boston Bowel Preparation Score (2)).  Monthly audits to ensure proceduralist compliance with recording of bowel preparation quality, with results provided to individual proceduralists for quality improvement.  Quarterly audits to summarise the proportion of colonoscopies with a poor bowel preparation.  Further education to patients that need a repeat procedure on how to achieve an appropriate preparation prior to colonoscopy. |
| Intubation distance^1,2^ | *“Unadjusted rates for cecal intubation should be ≥95%.”* | To ensure adequate views of the entire colon during colonoscopy, the intubation distance should be at the caecum, terminal ileum or anastomosis, and be recorded on the colonoscopy report.  Incomplete colonoscopy increases the risk for missed cancer (3), and often require repeat procedure within 12 months. | Recording of the intubation distance in a colonoscopy report with photo evidence.  Monthly audits to ensure proceduralist compliance with recording of intubation distances, with results provided to individual proceduralists for quality improvement.  Quarterly audits to summarize the proportion of colonoscopies with an inadequate intubation distance. |
| Withdrawal time^1,2^ | *“Withdrawal times of more than 6 minutes for examinations without polypectomy are a surrogate marker for adenoma detection rates, but cannot be relied on as an independent quality indicator.”* | To ensure sufficient examination, colonoscopy withdrawal time should be longer than 6 minutes. | Recording of the withdrawal time in a colonoscopy report.  Monthly audits to ensure proceduralist compliance with recording of withdrawal times, with results provided to individual proceduralists for quality improvement.  Quarterly audits to summarize the proportion of colonoscopies with an inadequate withdrawal time. |
| Adenoma and serrated polyp detection rate^2,3^ | *“Individual proceduralists should routinely document and maintain their adenoma detection rate at >25% in patients over 50-years of age in patients without a diagnosis of inflammatory bowel disease.”*  *“Serrated polyp detection rates are likely to be an equally valid marker of quality as adenoma detection rates and increasing evidence suggests that maintaining a rate of >10% in patients over the age of 50 without a diagnosis of inflammatory bowel disease may be a suitable indicator.”* | Several studies have provided evidence that lower adenoma detection rates are associated with increased risk for missed cancer or advanced lesion at colonoscopy (4, 5).  Calculating adenoma and serrated polyp detection rates is typically labor intensive and is not always reported appropriately for each proceduralist. | Recording of histopathology for each lesion found at each colonoscopy against each proceduralist, alongside procedural indications to identify those without inflammatory bowel disease.  Monthly audits to calculated adenoma and serrated polyp detection rates, with results fed back to the individual doctor to allow for quality improvement practice.  Quarterly audits to summarize the proportion of proceduralists with adenoma and serrated polyp detection rates above the international benchmark of 25% and 10% respectively.  Transparent results so that consumers and family physicians can request proceduralist performance statistics. |
| Colonoscopy complications | *“Perforation rates post colonoscopy should be <1/1,000.”* | Rates of procedure-related complications should be monitored and reported. | All complications that could be associated with the procedures and occur within a set timeframe of the colonoscopy should be recorded within the clinical data collection, such as perforation, post-polypectomy bleed, and death.  Six-monthly audits for summarized findings per hospital should be reported. |
| Post-colonoscopy colorectal cancer^4^ | Colorectal cancer after a negative colonoscopy should be minimized through good quality procedures. | An “interval cancer” is one that occurs within 5 years of a negative colonoscopy. A conservative estimate for the number of interval cancers is 1 per 1000 colonoscopies (6). | Ideally, data linkage should be made with the state cancer registry, as well as with histopathology reporting systems to automate new diagnoses of colorectal cancers occurring in existing patients.  Analysis and reporting should be undertaken for each occurrence of an interval cancer to understand the possible causes and future mitigation strategies.  Six-monthly audits for summarized findings per hospital should be reported. |
| Timely procedures^1^ | Surveillance colonoscopies should be completed within a timeframe recommended by the health network. | A delay in completion of surveillance colonoscopy increases risk for development of colorectal cancer.  The timeframe for completion of surveillance colonoscopy ranges from within 3-6 months of the national guideline recommended due date. | An appropriate computerized recall system should arrange for surveillance colonoscopies to be booked within an appropriate timeframe.  Quarterly audits for the proportion of surveillance colonoscopies being done later than recommended per hospital should be reported. |
| Reporting time^5^ | Appropriate and timely communication with patients. | Poor communication related to surveillance may cause patient anxiety or poor maintenance within the surveillance program. | Colonoscopy findings and other related communications should be provided in a format and timeframe that is suitable for the patient, while following best practice recommendations.  Communication strategies should be co-designed with consumer input. |
| Patient-reported measures^6^ | Ensure that partnerships are meeting the need of consumers | The collection of patients reported measures will ensure that the patient voice is part of the program. | Appropriate patient-reported measures should be decided in consultation with end-users.  Patient-reported measures should be regularly collected before and after the colonoscopy.  Specific feedback should be reported back to clinical teams monthly.  Aggregated data will be used as a measure of service quality, drive continuous improvement, and inform value-based healthcare models. |
| ^1^Colonoscopy Clinical Care Standard 4 (7); ^2^ Quality indicators for colonoscopy (8); ^3^Colonoscopy Clinical Care Standard 6 (7); ^4^Interval Colorectal Cancer After Colonoscopy (6); ^5^Colonoscopy Clinical Care Standard 9 (7); ^6^NSQHS Partnering with Consumers Standard (9); | | | |

**Supplementary Table S2.** Examples of risk factors as included in guidelines on surveillance colonoscopy in Australia (10, 11).

| Risk factor | Considerations and timing of surveillance colonoscopy |
| --- | --- |
| Colorectal neoplasia | - Findings from the previous two colonoscopies are used unless the patient has a prior history of CRC. - Intervals range between 6 months to 10 years. - Intervals length is dependent on lesion characteristics at each colonoscopy. |
| Age and comorbidities | - Surveillance may not be needed in patients aged ≥75 years. - Consider use of the Charlson comorbidity index when considering surveillance colonoscopy. |
| Family history of CRC | - The need for surveillance depends on number of family members affected and the degree of relation. |
| Hereditary CRC syndrome (e.g. Lynch syndrome) | - Interval length is 1-2 years. |
| CRC; colorectal cancer | |

**Supplementary Table S3.** Power summary for guideline concordance improvement under the stepped-wedge cluster randomized trial (SW-CRT) design

| **Effect size (Absolute)** | **Baseline concordance** | **Target concordance** | **Relative increase** | **Power** |
| --- | --- | --- | --- | --- |
| 5.0% | 60.0% | 65.0% | 8.3% | 80.0% |
| 6.0% | 60.0% | 66.0% | 10.0% | 90.0% |
| 7.0% | 60.0% | 67.0% | 11.7% | 100.0% |
| 8.0% | 60.0% | 68.0% | 13.3% | 100.0% |
| 9.0% | 60.0% | 69.0% | 15.0% | 100.0% |
| 10.0% | 60.0% | 70.0% | 16.7% | 100.0% (10% increase) |
| 11.0% | 60.0% | 71.0% | 18.3% | 100.0% (10% increase) |
| 12.0% | 60.0% | 72.0% | 20.0% | 100.0% |
| 13.0% | 60.0% | 73.0% | 21.7% | 100.0% |
| 14.0% | 60.0% | 74.0% | 23.3% | 100.0% |
| 15.0% | 60.0% | 75.0% | 25.0% | 100.0% (Target effect) |
| 16.0% | 60.0% | 76.0% | 26.7% | 100.0% |
| 17.0% | 60.0% | 77.0% | 28.3% | 100.0% |
| 18.0% | 60.0% | 78.0% | 30.0% | 100.0% |
| 19.0% | 60.0% | 79.0% | 31.7% | 100.0% |
| 20.0% | 60.0% | 80.0% | 33.3% | 100.0% (20% increase) |
| 21.0% | 60.0% | 81.0% | 35.0% | 100.0% |
| 22.0% | 60.0% | 82.0% | 36.7% | 100.0% |
| 23.0% | 60.0% | 83.0% | 38.3% | 100.0% |
| 24.0% | 60.0% | 84.0% | 40.0% | 100.0% |
| 25.0% | 60.0% | 85.0% | 41.7% | 100.0% |

Assuming an intraclass correlation coefficient (ICC) of 0.010, a baseline guideline concordance rate of 60%, and an expected post-implementation improvement to 75% (a 15% absolute increase), the study design provides approximately 100% power at a two-sided significance level of α = 0.05 under a binomial mixed-effects model. With a total of 26,775 colonoscopy episodes across five local health networks over seven periods (21 months in total), the study is adequately powered to detect the targeted 15% increase in concordance, confirming its feasibility for evaluating the intervention’s impact.


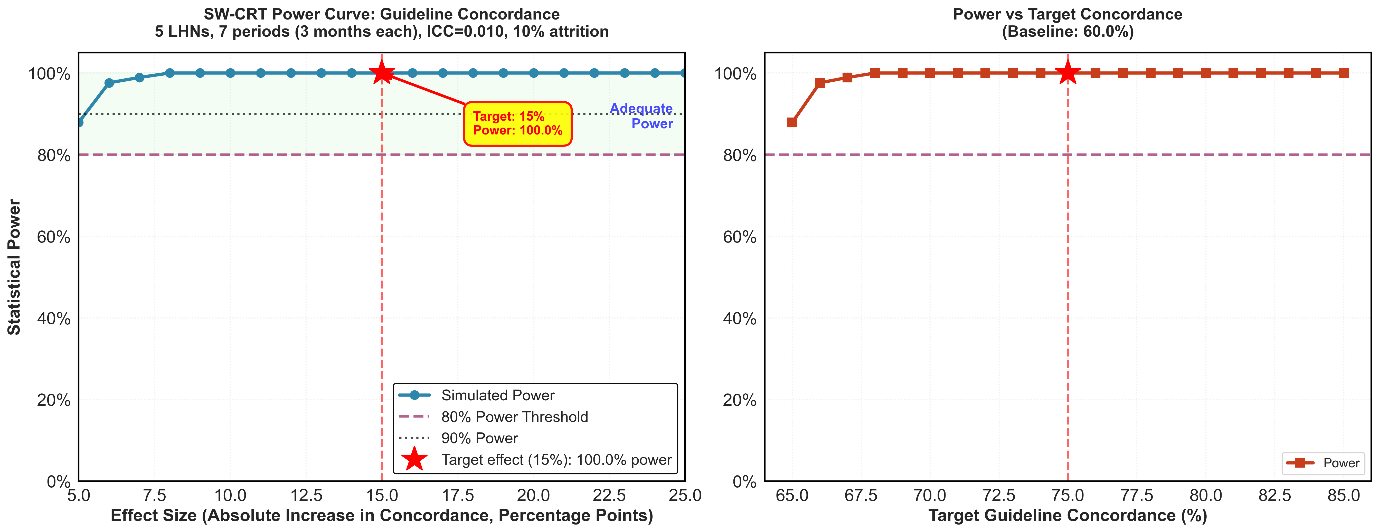


**Supplementary Figure S1:** Power curve for the stepped-wedge cluster randomized trial (SW-CRT) evaluating improvement in guideline concordance.

The plot illustrates the statistical power (y-axis) as a function of the expected improvement in guideline concordance (x-axis). The design assumes five local health networks (clusters) contributing to eight hospitals, with seven 3-month period (one baseline and six rollout periods). Power estimation assumes a baseline concordance of 60%, an intraclass correlation coefficient of 0.010, and an average of 1,417 colonoscopy episodes per period after 10% attrition. Power was estimated using a simulation-based approach in Python 3.9 (*statsmodels*), employing logistic mixed-effect model with cluster random intercepts and fixed effects to approximate the binomial outcome structure. Each point represents the Mean of 1000 simulation iterations per effect size. The horizontal dashed line indicates the conventional 80% power threshold. Under these assumptions, a 15% absolute improvement (60% to 75%) achieves approximately 90% power at a two-sided =0.05, confirming that the planned sample size (26,775 colonoscopy episodes) and stepped design are statistically adequate for detecting the target effect.


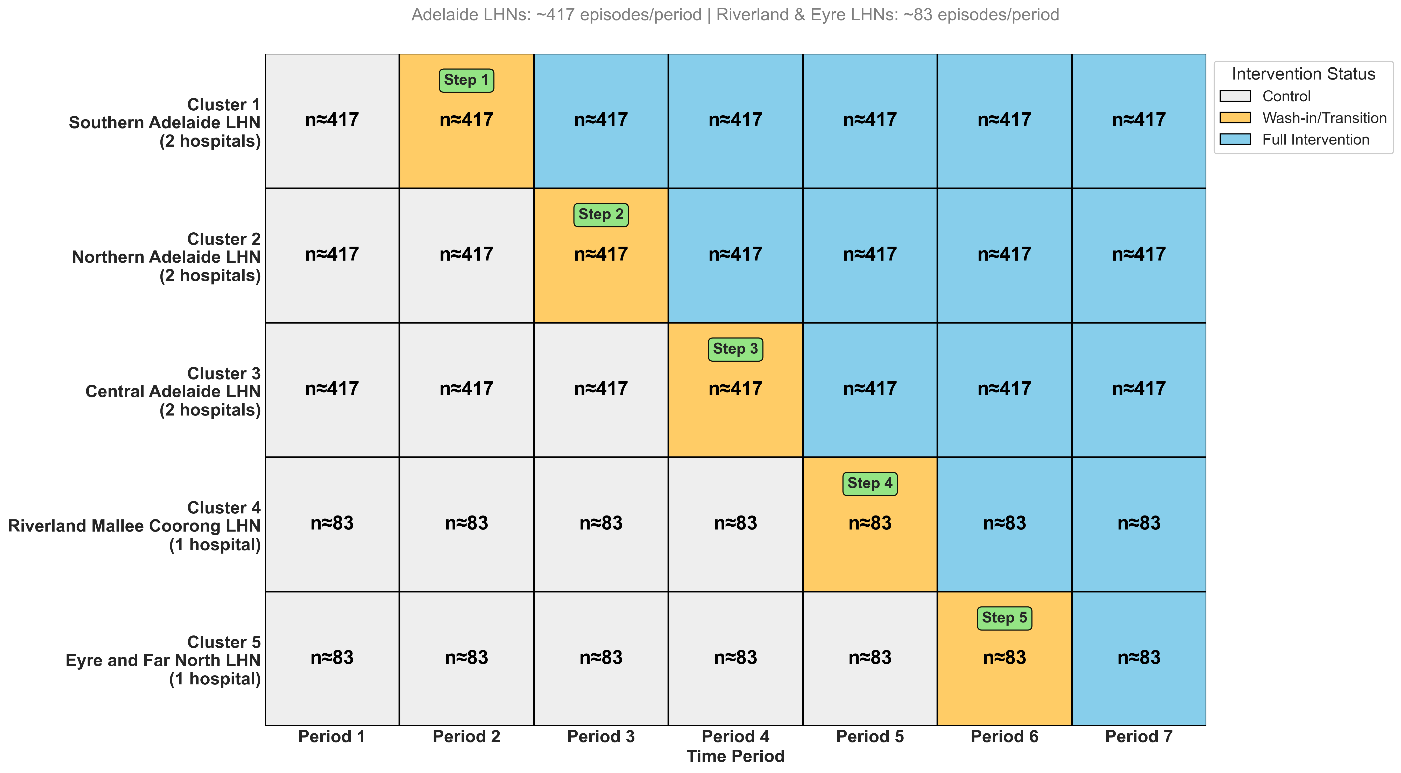


**Supplementary Figure S2**: Stepped-Wedge Cluster Randomized Trial Schematics.

The diagram illustrates the SW-CRT design of 5 local health networks (clusters), comprising a total of 8 participating hospitals, across 7 time periods (1 baseline and 6 rollout steps). Each time period contributes a total of approximately 1,417 colonoscopy episodes to the analysis (aggregated across all clusters and adjusted for 10% attrition). This total is composed of the three large local health networks (contributing 417 episodes each) and two smaller local health networks (contributing 83 episodes each) per period. Each cell represents a cluster-period, with light grey indicating control, gold/yellow indicating the transition (wash-in) period, and sky blue indicating full intervention, as shown in the legend. Step numbers (Step 1 to Step 5) annotate the randomized sequence of cluster transitions, starting from Period 2, with all clusters receiving the intervention by Period 7. Allocation concealment will be maintained by having an independent statistician generate the random sequence for cluster transitions, which will remain undisclosed to the research team until the rollout stage. A three-month wash-in period is planned to allow sufficient time for the intervention to be implemented and integrated before its effects are evaluated. To minimize the risk of contamination, outcome assessors will be blinded to the intervention status of each cluster, and all outcome data will be obtained from administrative systems that operate independently of the study team.

**References**

1. Hong SN, Sung IK, Kim JH, Choe WH, Kim BK, Ko SY, et al. The Effect of the Bowel Preparation Status on the Risk of Missing Polyp and Adenoma during Screening Colonoscopy: A Tandem Colonoscopic Study. Clin Endosc. 2012;45(4):404-11.

2. Lai EJ, Calderwood AH, Doros G, Fix OK, Jacobson BC. The Boston bowel preparation scale: a valid and reliable instrument for colonoscopy-oriented research. Gastrointestinal endoscopy. 2009;69(3 Pt 2):620-5.

3. Shergill AK, Conners EE, McQuaid KR, Epstein S, Ryan JC, Shah JN, et al. Protective association of colonoscopy against proximal and distal colon cancer and patterns in interval cancer. Gastrointestinal endoscopy. 2015;82(3):529-37.

4. Corley DA, Jensen CD, Marks AR, Zhao WK, Lee JK, Doubeni CA, et al. Adenoma detection rate and risk of colorectal cancer and death. The New England journal of medicine. 2014;370(14):1298-306.

5. Kaminski MF, Wieszczy P, Rupinski M, Wojciechowska U, Didkowska J, Kraszewska E, et al. Increased Rate of Adenoma Detection Associates With Reduced Risk of Colorectal Cancer and Death. Gastroenterology. 2017;153(1):98-105.

6. Adler J, Robertson DJ. Interval Colorectal Cancer After Colonoscopy: Exploring Explanations and Solutions. The American journal of gastroenterology. 2015;110(12):1657-64; quiz 65.

7. Australian Commission on Safety and Quality in Health Care. Colonoscopy Clinical Care Standard. . In: Care ACoSaQiH, editor. Sydney: ACSQHC; 2020.

8. Rex DK, Schoenfeld PS, Cohen J, Pike IM, Adler DG, Fennerty MB, et al. Quality indicators for colonoscopy. Gastrointestinal endoscopy. 2015;81(1):31-53.

9. Australian Commission on Safety and Quality in Health Care. National Safety and Quality Health Service Standards. In: ACSQHC, editor. 2nd edition ed. Sydney2017.

10. Cancer Council Australia Colonoscopy Surveillance Working Party. Clinical Practice Guidelines for Surveillance Colonoscopy. Bell C, editor. Sydney: Cancer Council Australia,; 2018.

11. Cancer Council Australia Colorectal Cancer Guidelines Working Party. Clinical practice guidelines for the prevention, early detection and management of colorectal cancer. Sydney: Cancer Council Australia; 2018 [Available from: <https://wiki.cancer.org.au/australiawiki/index.php?oldid=191477>.
