# Supplementary material for: A Digital Health Approach to Improve Compliance With Surveillance Colonoscopy Guidelines: The SCOPES Program: Study Protocol for a Stepped‐Wedge Cluster Trial
Source: Cancer Med. 2026 Feb 3;15(2):e71456. doi: 10.1002/cam4.71456 (PMC12868931; doi:10.1002/cam4.71456)
Supplement: Supplementary file 2 — File S1: cam471456‐sup‐0002‐FileS1.docx. [file CAM4-15-e71456-s001.docx]

**Supplementary File: Colonoscopy Screening Interval Recommendation Algorithm**

**Version:** 1.0

**Date:** October 22, 2025

**Status:** Frozen for trial use

**DOI/Registry:** [To be assigned]

1. **Algorithm Overview**

This document provides complete pre-registered decision logic for automated colonoscopy surveillance interval recommendations based on colonoscopy findings, patient characteristics, and Australian guideline for colonoscopy criteria.

- 1. **Design Principles**
- **Rule-based cascade**: Conditions evaluated sequentially (1-15)
- **First-match wins**: Once a recommendation is assigned, subsequent conditions are skipped
- **Precedence order**: Urgent/high-risk conditions evaluated first
- **Default handling**: All cases receive a recommendation or flag for clinical review by Nurses.

1. **Decision Logic Flowchart**

START

START
 ├─► Condition 1: Adenocarcinoma detected?
 │ ├─ YES → "Surgical Follow-up" → END
 │ └─ NO → Continue
 │
 ├─► Condition 2: Incomplete removal / High-risk genetics?
 │ ├─ YES → "Specialist Review" → END
 │ └─ NO → Continue
 │
 ├─► Condition 3: Missing quality data / Biopsy only?
 │ ├─ YES → "Nurse to complete (bowel)" → END
 │ └─ NO → Continue
 │
 ├─► Condition 4: Poor quality colonoscopy (excluding surveillance indication) / Polyp not removed?
 │ ├─ YES → " 3 months" → END
 │ └─ NO → Continue
 │
 ├─► Condition 5: Large polyp (≥20mm) with concerning or missing shape?
 │ ├─ YES → "6 months" → END
 │ └─ NO → Continue
 │
 ├─► Condition 6: Poor quality colonoscopy (for surveillance indication)?
 │ ├─ YES → "12-month (Bowel)" → END
 │ └─ NO → Continue
 │
 ├─► Condition 7: High polyp burden (≥3 polyps with complexity)?
 │ ├─ YES → "12-month (Polyp)" → END
 │ └─ NO → Continue
 │
 ├─► Condition 8: Age 75-80 or >80?
 │ ├─ 75-80 + No neoplasia → "Not recommended (No neoplasa and age 75-80)" → END
 │ ├─ 75-80 + Other findings → "To be considered (Age 75-80)" → END
 │ ├─ >80 → "Not recommended (Age >80)" → END
 │ └─ NO → Continue
 │
 ├─► Condition 9: Moderate polyp burden (1-9 polyps with specific criteria)?
 │ ├─ YES → "3 years" → END
 │ └─ NO → Continue
 │
 ├─► Condition 10: Low-risk (1-2 small polyps, no metabolic syndrome)?
 │ ├─ YES → "10 years" → END
 │ └─ NO → Continue
 │
 ├─► Condition 11: Any adenoma or serrated polyp present?
 │ ├─ YES → "5 years (adenoma)" → END
 │ └─ NO → Continue
 │
 ├─► Condition 12: Significant family history?
 │ ├─ YES → "5 years (Family)" → END
 │ └─ NO → Continue
 │
 ├─► Condition 13: Benign polyp findings (inflammatory polyp, lipoma, hyperplastic)?
 │ ├─ YES → "No surveillance recommended" → END
 │ └─ NO → Continue
 │
 ├─► Condition 14: No polyps?
 │ ├─ YES → "No surveillance recommended" → END
 │ └─ NO → Continue
 │
 └─► Condition 15: Missing data or edge cases
 └─ "Nurse to complete" → END

END

END

END

END

END

END

END

END

END

END

END

END

END

END

END

1. **Specific examples.**
   1. **Age Considerations**

**Priority:** Medium

**Recommendation:** Variable based on age and findings

**Inclusion Criteria:**

**Age 75-80:**

- Outcome = "no neoplasia " where the recommendation interval is "Not recommended (No neoplasia and age 75-80)"
- Other findings given the age flagged as "To be considered (Age 75-80)"

**Age >80:**

- Any findings and focusing on age only, the recommendation is "Not recommended (Age >80)"

**NLP Rules:**

- Numeric age comparison: 75 <= age <= 80 or age > 80
- Substring match for outcome (case-insensitive)
- Age conversion: float (age) with error handling (default 0)

**Rationale:**

- Guidelines recommend against routine surveillance in elderly due to competing mortality risks
- Ages 75-80 require individualised assessment

**Examples:**

| **Scenario** | **Age** | **Outcome** | **Result** |
| --- | --- | --- | --- |
| 1 | 78 | "Normal" | Not recommended (Normal/benign and age 75-80) |
| 2 | 77 | "Tubular adenoma" | To be considered (Age 75-80) |
| 3 | 85 | "3 adenomas" | Not recommended (Age >80) |

- 1. **Three Years (Moderate Polyp Burden)**

**Priority:** Low-Medium

**Recommendation:** "3 years"

**Inclusion Criteria (num_polyps_patho ≥1 AND complex rules):**

**Detailed criteria:**

1. **3-4 polyps + <10mm:** TVA/VA with any dysplasia OR TA with high-grade dysplasia
2. **1-2 polyps + ≥10mm + TA**
3. **3-4 polyps + ≥10mm + TA without high-grade dysplasia**
4. **5-9 polyps + <10mm + TA without high-grade dysplasia**
5. **1-2 polyps + ≥10mm:** SSL or dysplasia or TSA
6. **3-4 polyps, all SSL, all <10mm, no dysplasia, no TSA**
7. **3-9 polyps, all SSL/TA with no dysplasia and <10mm**
8. **2-4 polyps:** (SSL ≥10mm or SSL with dysplasia) OR (TA <10mm no dysplasia) OR TSA
9. **2 polyps:** ≥1 SSL + ≥1 TVA/VA
10. **Any polyp 10-20mm** (inclusive)

**NLP Rules:**

- Size comparisons: <10, ≥10, 10-20 ranges
- Pathology exact matches
- Dysplasia level checks
- Counting logic for polyp types

**Examples:**

| **Scenario** | **Num Polyps** | **Pathology** | **Size (mm)** | **Dysplasia** | **Result** |
| --- | --- | --- | --- | --- | --- |
| 1 | 2 | TA | 12 | Low grade | 3 years |
| 2 | 4 | SSL | 8 | No dysplasia | 3 years |
| 3 | 1 | TA | 15 | Low grade | 3 years |

- 1. **Default Nurse Recommendation**

**Priority:** Lowest (catch-all)

**Recommendation:** "Nurse recommendation"

**Inclusion Criteria (ANY of):**

1. **Empty indication:** Indication field is empty
2. **Assessment indications:** Indication contains "surgical assessment" or "completion scope"
3. **Missing shape for large polyp:** Shape is empty AND size ≥20mm
4. **Polyp not retrieved:** Outcome contains "polyp not retrieved"

**NLP Rules:**

- Empty check: str().strip() == ""
- Substring match for indications (case-insensitive)
- Numeric size comparison

**Rationale:**

- Catch-all for incomplete data or edge cases
- Triggers manual review by nursing staff

**Examples:**

| **Scenario** | **Indication** | **Shape** | **Size (mm)** | **Outcome** | **Result** |
| --- | --- | --- | --- | --- | --- |
| 1 | "" | "Sessile" | 5 | "Normal" | Nurse recommendation |
| 2 | "Surgical assessment" | "Flat" | 10 | "Normal" | Nurse recommendation |
| 3 | "Symptoms" | "" | 25 | "Complete" | Nurse recommendation |
| 4 | "Screening" | "Sessile" | 5 | "Polyp not retrieved" | Nurse recommendation |

1. **NLP Pipeline Documentation**
   1. **Negation Handling**

**Current Implementation:**

- **No negation detection** for adenocarcinoma (Condition 1)
  - Rationale: Conservative approach for malignancy
  - Any mention triggers follow-up
- **Absence-based negation** for intubation (Condition 4)
  - Checks for presence of success terms: "caecum", "anastomosis", "terminal ileum"
  - Absence = failed intubation

**Ambiguous Cases:**

| **Text** | **Interpretation** | **Rationale** |
| --- | --- | --- |
| "No adenocarcinoma" | Triggers follow-up | Conservative: pathologist reviewed case |
| "Rule out adenocarcinoma" | Triggers follow-up | Uncertainty requires follow-up |
| "Previous adenocarcinoma" | Triggers follow-up | History relevant for surveillance |
| "Caecum not reached" | Failed intubation | Absence of success terms |

**Future Enhancement Considerations:**

- Implement NegEx algorithm for proper negation detection
- Add temporal qualifiers (previous vs current)
- Uncertainty detection ("rule out", "possible")
  1. **Synonym Handling**

**Pathology Synonyms:**

- Tubular adenoma: TA
- Tubulovillous adenoma: TVA
- Villous adenoma: VA
- Sessile serrated lesion: SSLP, sessile serrated adenoma, SSA, sessile serrated polyp (SSP)
- Traditional serrated adenoma: TSA
- Familial adenomatous polyposis: FAP
- MUTYH-associated polyposis: MAP

**Genetic Condition Synonyms:**

- Lynch syndrome: Lynch, hereditary nonpolyposis colorectal cancer, HNPCC
- FAP: familial adenomatous polyposis, adenomatous polyposis coli, APC
- Serrated polyposis syndrome: hyperplastic polyposis syndrome

**Bowel Preparation Quality:**

- Poor: "poor", "inadequate", "suboptimal" (all matched as "poor")
- Good: "good", "adequate", "satisfactory"
- Excellent: "excellent", "optimal"

1. **Algorithm Versioning**
   1. **Version History**

| **Version** | **Date** | **Changes** | **Status** |
| --- | --- | --- | --- |
| 0.9-beta | 2025-02-15 | Initial development | Testing |
| 0.95 | 2025-08-01 | Added benign pathology handling | Pre-registration |
| **1.0** | **2025-10-22** | **Fixed condition checks, added Condition 13.5** | **Frozen for trial** |

- 1. **Version 1.0 Specifications**

**Frozen Date:** October 22, 2025

**Trial Registration:** ACTRN12624001429549

**Critical Fixes in v1.0:**

1. Changed if index not in recommendations: to if pd.notna(recommendations[index]): to properly check assigned recommendations
2. Added Condition 13.5 for benign non-polyp findings
3. Updated Condition 4 to include "polyp/s - not retrieved"
4. Updated Condition 14 to handle "Surgical follow up" with zero polyps
5. Fixed Condition 11 column name from {i}-size (mm) to {i}-size_numeric

**No changes permitted during trial period without version increment.**

- 1. **Post-Trial Update Process**

**For non-breaking changes (v1.1, v1.2, etc.):**

- Additional synonyms
- New benign pathology terms
- Clarifications to existing logic
- Bug fixes not affecting recommendation assignments

**For breaking changes (v2.0):**

- Changes to condition precedence
- New conditions inserted mid-cascade
- Changes to inclusion/exclusion criteria
- Modifications to NLP logic affecting matching

**Update Protocol:**

1. Document proposed change with clinical rationale
2. Test on validation cohort
3. Compare outputs v1.0 vs new version
4. Update this supplementary file
5. Increment version number appropriately
